# Recent Progress in the Synthesis of Drugs and Bioactive Molecules Incorporating Nitro(het)arene Core

**DOI:** 10.3390/ph15060705

**Published:** 2022-06-03

**Authors:** Maxim Bastrakov, Alexey Starosotnikov

**Affiliations:** N.D. Zelinsky Institute of Organic Chemistry RAS, Leninsky Prosp. 47, Moscow 19991, Russia; alexey41@list.ru

**Keywords:** nitro group, arenes, heterocycles, drugs, biological activity

## Abstract

Aromatic nitro compounds play a unique role in the synthesis of drugs and pharmaceutically oriented molecules. This field of organic chemistry continues to be in demand and relevant. A significant number of papers are published annually on new general methods for the synthesis of nitrodrugs and related biomolecules. This review is an analysis of the literature on methods for the synthesis of both new and already-known aromatic and heteroaromatic nitrodrugs covering the period from 2010 to the present.

## 1. Introduction

The synthesis and study of biologically active compounds remains one of the most important and developing areas of organic and medicinal chemistry. Aromatic nitro compounds of both natural and synthetic origin constitute a broad class of organic molecules that exhibit a wide range of biological activities and are being used as drugs [1,2]. The spectrum of activity directly depends on the nature and mutual arrangement of substituents in a molecule. A significant number of papers are published annually not only on toxicity and metabolism of nitrodrugs and related biomolecules, but also on new general methods for their synthesis [3,4,5,6]. These studies are driven by the need to reduce costs and environmental impact during industrial production.

This review is an analysis of the literature on methods for the synthesis of both new and previously known aromatic and heteroaromatic nitrodrugs covering the period from 2010 to the present.

## 2. Nitrobenzene Derivatives

**Scheme 1:**
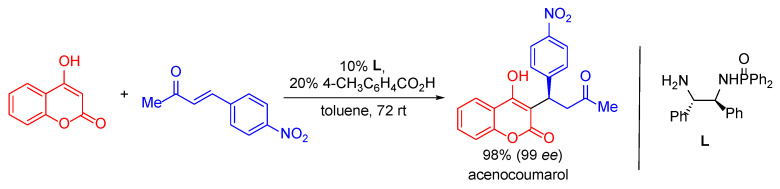
Synthesis of enantiomerically pure (*R*)-acenocoumarol.

**Acenocoumarol** is an anticoagulant that acts as a vitamin K antagonist (similar to warfarin). It is a coumarin-based generic drug and is sold under many brand names around the world. Michael addition of 4-hydroxycoumarin to α,β-unsaturated ketones is a straightforward method to access warfarin analogues. Basically, acenocoumarol has been prescribed as racemate; however, both enantiomers are also described. Each of them demonstrates different activity and metabolism. In the last few years, a number of new methods for the syntheses of acenocoumarol as well as other nitro-containing warfarin derivatives using organocatalysis have been published, leading to enantiomerically pure Michael adducts [7] (Scheme 1).

Other organocatalysts can be used in these reactions, such as 2-amino-DMAP/prolinamide [8], α-helical peptide foldamer [9], enantiomerically pure (S,S)-diphenylethylenediamine [10], and binaphthyl-modified 1,2-diphenylethylenediamine [11]. In addition, Vaccaro et al. proposed a method for the synthesis of warfarin derivatives (including acenocoumarol) using polystyrene-supported 1,5,7-triazabicyclo[4.4.0]dec-5-ene (TBD) as a catalyst [12].

**Scheme 2:**
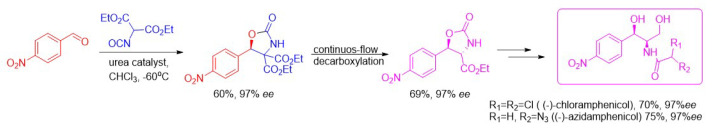
Synthesis of amphinicol derivatives.

Antibiotics of the **amphinicol** group have been of interest to researchers for several decades due to their wide spectrum of activities against Gram-positive and Gram-negative bacteria. Among the representatives of the amphinicol group, there are nitrogroup-containing compounds. Recently, Chen et al. proposed two methods for the synthesis of **azidamphinicol** and **chloramifinicol**. The reactions proceed with high stereo- and enantioselectivity. The first method is based on two key steps: urea-catalyzed aldol condensation of *p*-nitrobenzaldehyde and isocyanatomalonic ester leading to chiral oxazolidinone. Further decarboxylation of this compound occurs in a continuous flow reactor with the formation of *trans*-monoester of oxazolidinone with two adjacent stereocenters, which leads to *syn*-vicinal amino alcohols of the amphinicol family [13] (Scheme 2).

The key step of the second method is the Henri reaction catalyzed by the copper(II)-chiral biphenyl-substituted amino alcohol complex, which ultimately leads to 2-amino-1,3-diol, which undergoes multistep continuous-flow transformations, which also afford enantiomerically pure antibiotics of the amphinicol group [14].

The biosynthesis of chloramphinicol is another modern approach to this antibiotic. In 2012 and 2014, two research groups independently published articles on the biosynthesis of chloramphinicol. The key step in biosynthetic transformations is the enzyme-catalyzed N-oxidation of the amino group [15,16].

**Scheme 3:**
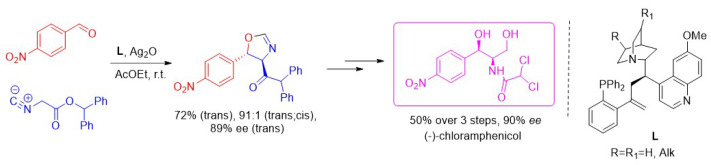
Dixon’s synthesis of (-)-chloramphenicol.

A four-step method for the synthesis of (-)-chloramphenicol was described by J. Dixon. It is based on the enantio- and diastereoselective aldol reaction of isocyanoacetates, with *p*-nitrobenzaldehyde catalyzed by Ag_2_O and aminophosphine ligands [17] (Scheme 3).

**Dantrolene** is a muscle relaxant, the mechanism of action of which is based on the blockade of ryanodine receptors (calcium channels of the sarcoplasmic reticulum of myocytes). This drug has been used since 1973 [18]. The original patent synthesis started with *p*-nitroaniline, which undergoes diazotization followed by a copper(II) chloride catalyzed arylation with furfural (essentially a modified Meerwein arylation). This then reacts with 1-aminohydantoin to form the final product [19].

**Scheme 4:**
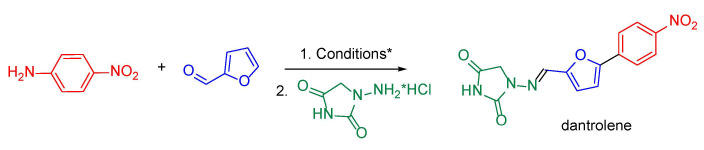
Synthesis of dantrolene. *—see Table 1.

The current literature describes several new methods for the multigram-scale synthesis of dantrolene [20,21,22]. The authors proposed new catalytic systems at the arylation step. In this case, both *p*-nitroanaline or *p*-nitrohalogenobenzenes can be used as starting compounds (Scheme 4, Table 1).

**Scheme 5:**
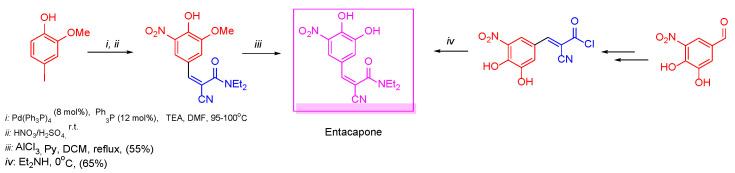
Fu’s and Srikanth’s methods for the synthesis of entacapone.

**Entacapone** is a nitrocatechol derivative and an inhibitor of catechol-O-methyltransfarase, and is used in the treatment of Parkinson’s disease as an adjunct to levodopa/carbidopa therapy. It was developed by Orion Pharma and is marketed by Novartis under the trade name Comtan in the United States. To date, several approaches to entacapone have been described. Harisha et al. described its synthesis from 2-cyano-3-(3-hydroxy-4-methoxy-5-nitrophenyl)prop-2-eneamide using amine-mediated demethylation [23,24]. An industrial method for the synthesis of entacapone via a Knoevenagel reaction of 2-cyano-3-(3-methoxy 4-hydroxy-5-nitrophenyl)acrylic acid was reported by Guo [25]. Fu and coworkers proposed another method starting from *p*-vanillin [26]. Srikanth et al. [27] reported on the condensation of 3,4-hydroxy-5-nitrobenzaldehyde with 2-cyanoacetic acid (Scheme 5).

In addition, preparation of entacapone from 4-iodo-2-methoxyphenol with 2-cyano-N,N-diethylacrylamide using a Pd-catalyzed Heck reaction as a key step was described [28].

**Scheme 6:**
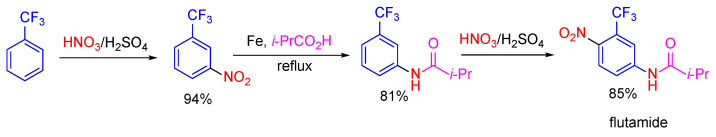
Synthesis of flutamide.

**Flutamide** (4-nitro-3-trifluoromethylisobutyranilide) is a nonsteroidal antiandrogen (NSAA) that is used primarily to treat prostate cancer. It is also used in the treatment of androgen-dependent conditions such as acne, excessive hair growth, and high androgen levels in women. In addition to previously published studies [29,30,31,32], a convenient and economically beneficial method was developed by S. Rahbar et al. [33] (Scheme 6). According to the procedure, benzotrifluoride was first nitrated, and the product was reduced and acylated in one pot in the presence of iron powder and isobutyric acid to produce 3-trifluoroisobutyranilide. Finally, flutamide was produced via further nitration.

General methods for the synthesis of structurally close arylamides using metal complex catalysis have been described recently [34,35]. According to the proposed methods, it is possible to synthesize 3-trifluoroisobutyranilide, a key intermediate in the synthesis of flutamide, in high yields.

Another synthesis of flutamide was described in 2016 by Ren et. al. [36]. The target compound was synthesized via trifluoromethylation of the corresponding nitro compound.

**Scheme 7:**
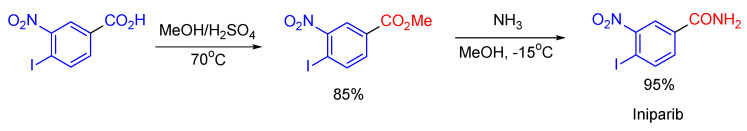
Scheme of the synthesis of iniparib.

**Iniparib,** a drug for cancer treatment, has been known since 2009. In 2013, its synthesis was published by Divi et al. [37] (Scheme 7). It is noteworthy that the proposed scheme allowed the researchers to obtain the product with virtually no impurities.

Later, the final step of this synthetic scheme was optimized in a patent [38].

**Scheme 8:**
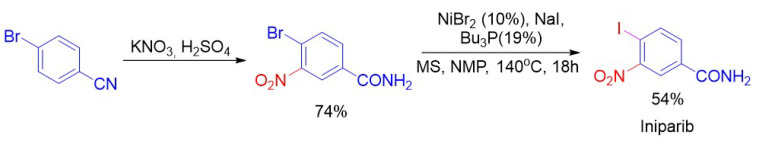
Synthesis of iniparib from 4-bromobenzonitrile.

In addition, a two-step synthesis of iniparib based on 4-bromobenzonitrile was described recently. The key step of the proposed synthesis was the iodination of the corresponding aryl bromide using metal complex catalysis [39] (Scheme 8).

The interest of researchers in dihydropyridine derivatives (including those containing nitro) has not weakened to date due to new types of activities revealed: anticonvulsant [40], antioxidant, anti-inflammatory, and antiulcer [41]. **Nifedipine** is a calcium-channel blocker of the dihydropyridine type that has been used since the last century [42] as a medicine for the treatment of diseases such as angina, high blood pressure (including during pregnancy), Raynaud’s phenomenon, and premature labor.

**Scheme 9:**
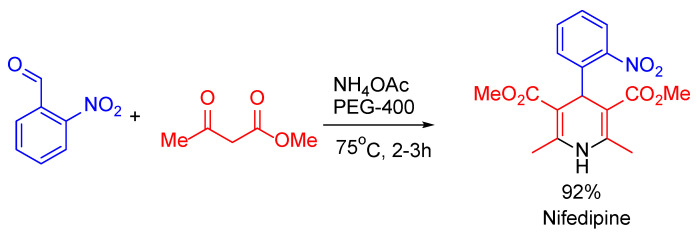
Modified Hantzsch synthesis of nifedipine.

New methods for assembling the dihydropyridine ring are regularly published; these can be used in the synthesis of nifedepine. For example, Siddaiah et al. published a modified Hantzsch PEG-mediated, catalyst-free synthesis under solvent-free conditions [43] (Scheme 9).

In addition, methods for the synthesis of nifedipine using new composite catalysts [44] and ionic liquids [45] were described.

**Scheme 10:**
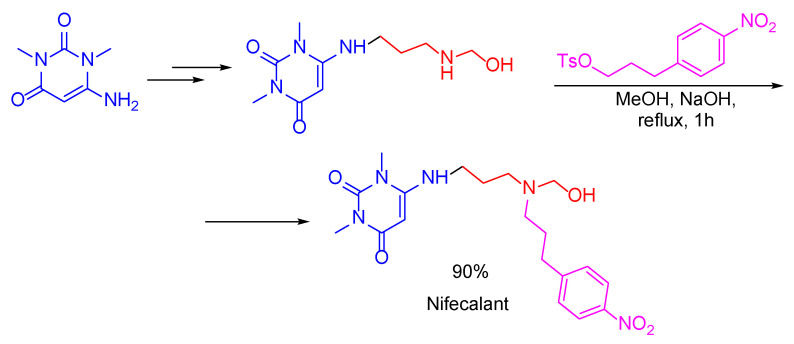
A method for the synthesis of nifecalant by Yi.

**Nifekalant** is a class III antiarrhythmic agent approved in Japan for the treatment of arrhythmias and ventricular tachycardia. Earlier, a method for the synthesis of nifecalant hydrochloride using dimethylurea as a starting material was described [46]. This method had a number of disadvantages: low yield (about 30%), high cost, and the use of corrosive reagents such as phosphorus oxychloride and sodium hydride in the preparation; these are harmful to the environment and are not conducive to industrial production. In 2013, Yi et al. published a new synthetic method on an industrial scale to produce nifecalant based on 6-amino-1,3-dimethyluracil. This method features a high yield and purity of the product, simple operation, and environmental tolerance of the used reagents [47] (Scheme 10).

**Scheme 11:**
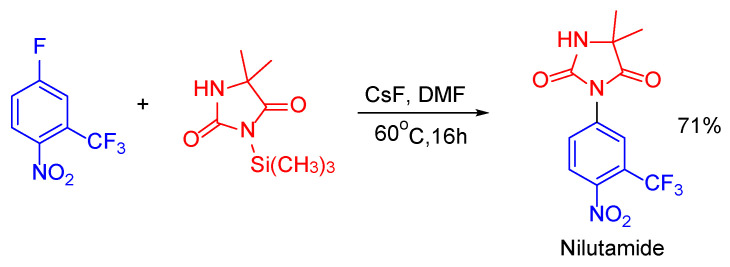
Synthesis of nilutamide.

**Nilutamide** is a nonsteroidal antiandrogen (NSAA) that is used in the treatment of prostate cancer. It has also been studied as a component of feminizing hormone therapy for transgender women and to treat acne and seborrhea in women. Nilutamide was first described in 1977 and introduced for medical use in 1987 in France [48]. However, interest in this compound has not weakened, even today. In 2010, a general method for the synthesis of nilutamide was published (this patent was later republished in 2021) via a reaction of haloarene with *N*-trimethylsilyl imidazoline-1,3-dione [49] (Scheme 11).

According to the authors of the patent, this method is simpler, faster, and cheaper to implement; provides high yields; and avoids toxic heavy-metal contamination compared to the methods described earlier.

**Scheme 12:**
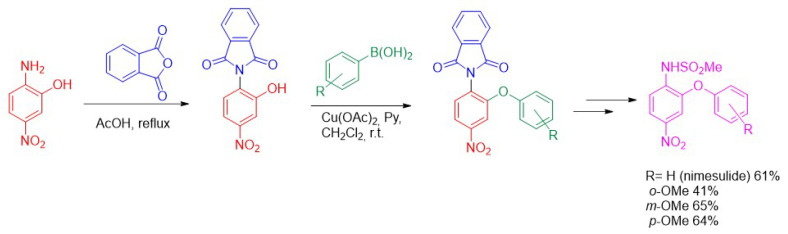
Synthesis of nimesulide and its analogs.

N-(4-nitro-2-phenoxyphenyl)methanesulfonamide (**nimesulide**) is a well-known brain cyclooxygenase (COX) inhibitor with increased selectivity for COX-2, which was reported to play a role in the physiological control of synaptic plasticity and neurological disorders, including cerebrovascular and neurodegenerative disorders such as Alzheimer’s and Parkinson’s diseases. In 2010, analogs of nimesulide containing methoxy substituents in the phenyl ring were designed, synthesized, and evaluated for potential as radioligands for brain COX-2 imaging. The synthesis of nimesulide analogs was based on the copper-mediated arylation of phenolic derivatives [50] (Scheme 12).

In vitro inhibition studies using a colorimetric COX (ovine) inhibitor-screening assay demonstrated that methoxy analogs of nimesulide also demonstrated an inhibition ability toward the COX-2 enzyme.

**Scheme 13:**
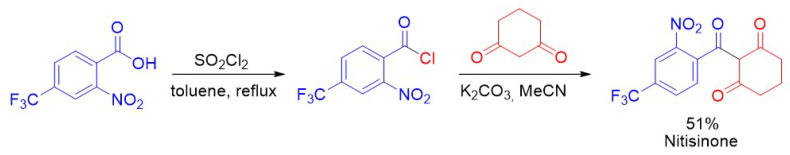
Improved method for the synthesis of nitisinone.

One more nitro-group-containing drug is 2-(2-nitro-4-trifluoromethylbenzoyl)-1,3-cyclohexanedione, also known as **nitisinone** or NTBC. NTBC is used to slow the effects of hereditary tyrosinemia type 1 (HT-1) in adult and pediatric patients. It was approved by the FDA and EMA in January 2002 and February 2005, respectively. Unfortunately, one of the problems of the actual drug formulation (i.e., Orfadin^®^ capsules) is its chemical instability. After opening, the drug can be used only for 2 months and must be stored at a temperature not exceeding 25 °C. This negatively affects the cost of the drug. In 2016, an improved method for the synthesis and purification of NTBC was proposed that allows the target product to be obtained with a stability of at least 6 months [51] (Scheme 13). The extreme stability of the drug is associated with the high purity of the product. The synthesis was accomplished according to a known scheme; however, the authors changed the reaction conditions and the purification process of the target compound.

**Scheme 14:**
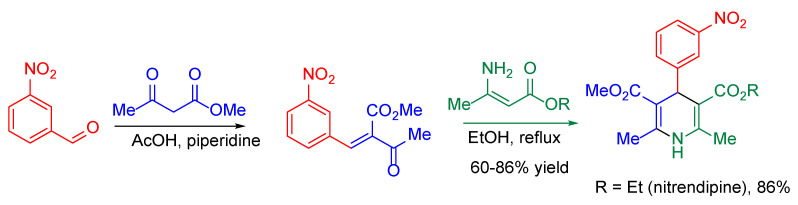
Synthesis of nitrendipine.

**Nitrendipine** is another representative of dihydropyridine derivatives used as a calcium-channel blocker. Recently, a number of studies of nitrendipine analogs have been carried out, including studies of their antihypertensive activity [52,53]. In 2011, Zhou et al. reported on the synthesis of nitrendipine analogs based on *m*-nitrobenzaldehyde. The transformations take place according to Scheme 14 below.

The synthesized compounds showed significant antihypertensive activity at the level of nitrendipine or higher.

**Scheme 15:**
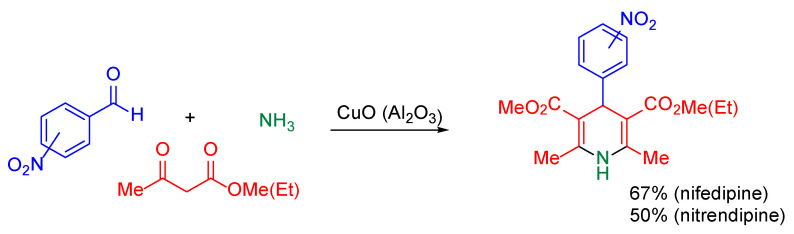
Modified Hantzsch synthesis of nifedipine and nitrendipne.

Modified synthesis of nifedipine and nitrendipne through a three-component Hantzsch reaction has been described under catalysis of either micellar heteropoly acids [54] or nanoscale metal oxides [55] (Scheme 15).

**Scheme 16:**
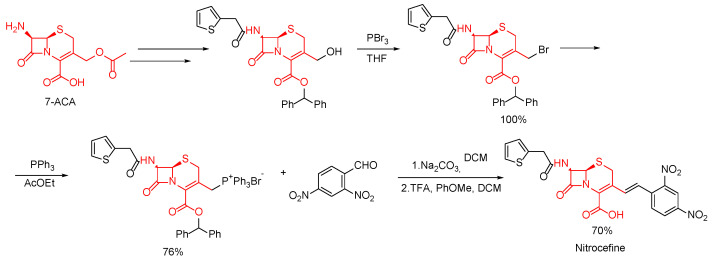
Wang’s synthesis of nitrocefin.

**Nitrocefin** is a cephalosporin that can be used to detect whether a bacteria produces beta-lactamase; i.e., to detect bacterial resistance. The detection of bacterial resistance can help to avoid overtreatment and treatment errors, thereby improving the efficiency and quality of treatment, reducing the suffering of patients, and reducing the cost of treatment. In 2016, Wang et al. [56] proposed an improved method for the synthesis of nitrocefin in seven steps by using 7-aminocephalosporanic acid (7-ACA) as a starting material (Scheme 16). 7-ACA is a key intermediate for cephalosporins and has been industrialized at a low market price. The new method has a simple operation, an easy reaction, convenient product purification, a high yield, easy industrialization, and a low production cost.

**Scheme 17:**
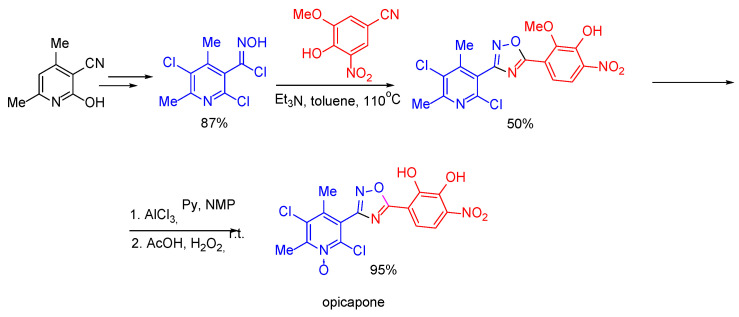
Synthesis of opicapone.

Another important nitro-containing drug described in the last decade is **opicapone**. This compound was approved for use in 2016, and is prescribed for people with Parkinson’s disease. Opicapon restores dopamine levels in the parts of the brain responsible for movement and coordination. The synthesis of opicapone and its intermediates has been recently described in a number of patents. Nabold et al. [57] published a new route for the synthesis of opicapone as shown in the following Scheme 17.

In addition, Sathe et al. patented a process for the preparation of opicapone that overcame the disadvantages of previously known methods [58].

Notably, the structural nitro analogs of opicapone were investigated as selective inhibitors of the catechol-O-methyltransferase (COMT) enzyme. They were found to have a reduced toxicity risk, and were endowed with a longer duration of inhibition than opicapone [59].

**Scheme 18:**
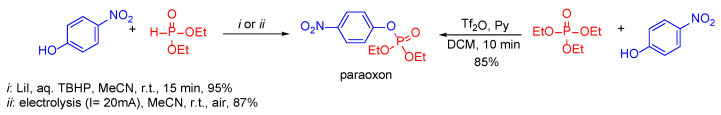
Methods for the synthesis of paraoxon.

Phosphate esters have been found in a variety of biological molecules, such as nucleic acids, proteins, carbohydrates, lipids, coenzymes and steroids. In this regard, new approaches to the synthesis of their derivatives are relevant and in demand. **Paraoxon** is one of the representatives of organophosphates; it acts as a cholinesterase inhibitor. During the last decade, several papers were published on new general methods of synthesis of organophosphates, including paraoxon. The key reactions are based on the interaction of phenols with phosphates and phosphites. The methods differ in the reaction conditions: electrolysis [60], a LiI/TBHP-mediated oxidative cross-coupling reaction [61], or interaction in the presence Tf_2_O/Py [62] (Scheme 18).

**Scheme 19:**
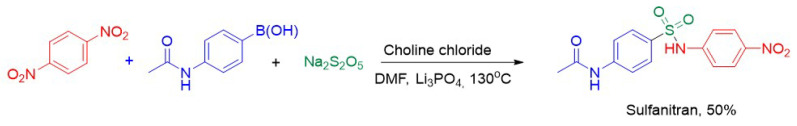
Jiang’s method for the synthesis of sulfanitran.

In 2020, Jiang et al. published a simple, convenient, and cheap approach to the construction of sulfonamides of various natures. This method is based on the direct sulfonamidation of the corresponding nitroarenes in the presence of boronic acids and sodium metabisulphite (Scheme 19). It is noteworthy that this method allows the quick and easy synthesis of pharmacologically oriented sulfonamides of both natural and synthetic origins, such as **sulfanitran**—a sulfonamide antibiotic actively used in the poultry industry to combat coccidioides [63].

## 3. Nitro Heterocycles

Along with the nitroarenes discussed above, nitroheterocyclic compounds constitute another important class of nitrodrugs. Basically, the nitroheterocyclic moiety causes broad-spectrum antibacterial, antiprotozoal, and antiparasitic activities. The widely recognized drugs nitrofural (5-nitro-2-furaldehyde semicarbazone) and metronidazole were initially developed in the 1940–1950s, and their numerous analogs possess an excellent balance between medicinal efficiency and chemical simplicity. However, further chemical modification of approved efficient nitrodrugs may result in a significant decrease in IC_50_ and/or toxicity values. This section encompasses recent advances in the synthesis of known drugs containing a nitroaromatic heterocyclic skeleton. The main classes of heterocycles considered are furans and imidazoles. In addition, some miscellaneous heterocyclic nitrodrugs of other classes will be discussed.

### 3.1. Nitrofurans

Nitrofurans represent a family of widely used antibacterial and antiparasitic drugs containing 5-nitro-2-hydrazonylfuran pharmacophore. However, they are usually associated with a variety of side effects such as hepatotoxicity or gastrointestinal disorders. The mechanisms that generate the cytotoxic effects of nitrofuran drugs are not yet clearly understood. The results obtained recently by Gallardo-Garrido and coworkers [64] suggest that the toxic effect of nitrofurans on mammalian cells is caused by the combined 2-hydrazonylfuran moiety, redox cycling of 5-nitrofuran, and inhibitory effects on antioxidant enzymes.

**Nitrofurantoin** (Furantin^©^) is an antibiotic generally used to treat urinary tract infections such as cystitis. Due to a poor solubility of nitrofurantoin in water, efforts have been made to prepare its cocrystals with a number of simple organic conformers: urea, nicotinamide, L-proline, 4-hydroxybenzoic, and citric and vanillic acids [65]. Among all synthesized cocrystals, only two of them were found to be stable in EtOH (urea and L-proline), and a cocrystal of nitrofurantoin with vanillic acid was stable in water. It was concluded that the significantly higher solubility of the conformers used provided cocrystals that were more soluble than the stable form of the drug in water.

**Scheme 20:**
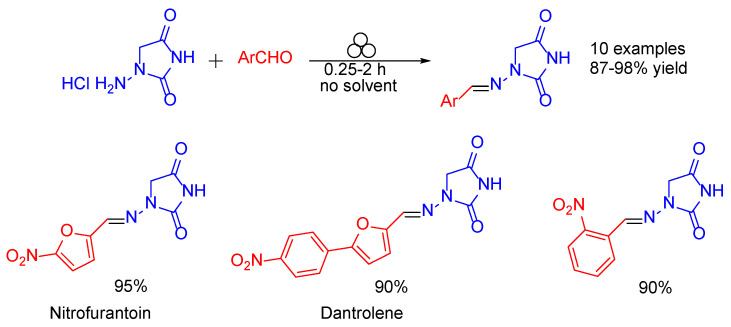
Gram-scale method for the preparation of nitrofurantoin and dantrolene.

The environmentally benign gram-scale method for the preparation of nitrofurantoin and the myorelaxant dantrolene (Dantrium^©^), as well as their structural analogs, was reported by Colacino and coworkers [66]. This novel mechanochemical procedure has a number of indisputable advantages: solvent-, base-, and waste-free; and high yields with no further purification required. In addition, the elaborated method allows the cost of the synthesis to be reduced considerably (Scheme 20).

**Scheme 21:**
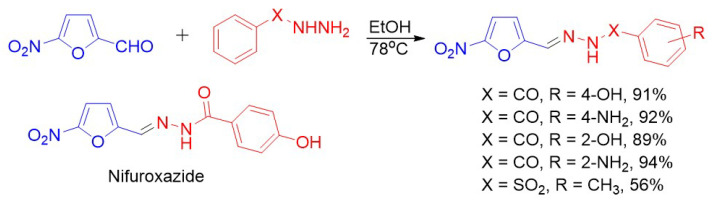
Synthesis of nifuroxazide analogs.

A number of structural analogs of the antimicrobial **nifuroxazide** were synthesized and tested for antimicrobial activity against a panel of bacteria and the pathogenic fungus Candida albicans [67]. Chemical diversity was achieved by shuffling the substituents in the furan and benzene rings. Most compounds showed activity against Gram-negative bacteria, while only 5-nitrofuran derivatives were active against Candida albicans, thus indicating the crucial role of the nitro group (Scheme 21).

A simple high-yield method for the synthesis of **nitrofural** (Furacilin) under the action of the supported catalyst CuO/CNTs has been patented [68]. An intermediate 5-nitrofurfural was synthesized from furfuryl alcohol used as a raw material through esterification, nitration, deprotection, and oxidation. Then, 5-nitrofurfural and semicarbazide were subjected to a condensation to give nitrofural. The authors positioned this method as simple to operate and the adopted catalyst as nontoxic, easy to remove, and renewable.

**Scheme 22:**
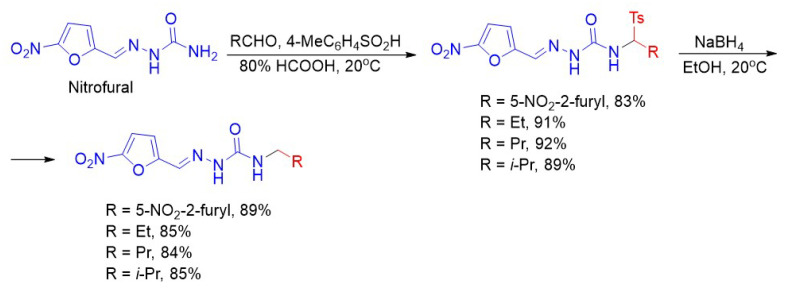
Synthesis of new bioactive molecules on the basis of nitrofural.

Application of nitrofural as a source of novel semicarbazones with potential antibacterial and antifungal activity was recently reported by Fesenko et al. [69]. Reactions of nitrofural with aldehydes and *p*-toluene sulfinic acid gave N(4)-substituted semicarbazones, which upon reduction with NaBH_4_ underwent tosyl group removal (Scheme 22).

The obtained compounds showed potent antifungal activity against *C. albicans* and *C. neoformans,* with MIC values of 8–32 μg/mL. They also possessed high antibacterial activity against Gram-positive *S. aureus* (MIC 8–16 μg/mL), Gram-negative *E. coli,* and *A. baumannii* (MIC 8–16 μg/mL).

**Scheme 23:**
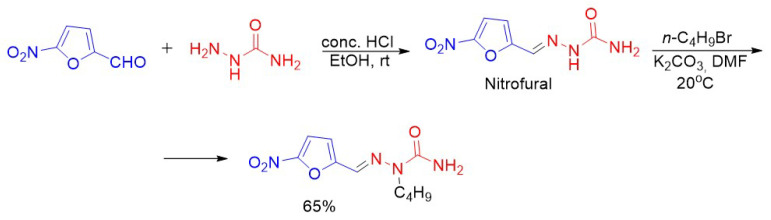
Synthesis of nitrofural derivative.

Arylsemicarbazones, including nitrofural and its (N2)-alkyl derivative, were identified as valuable compounds for the development of novel anticancer agents [70]. The synthesis was accomplished starting with 5-nitrofurfural and semicarbazide, followed by alkylation with *n*-butyl bromide (Scheme 23).

The N-butyl compound was found to be active against the K562, HL-60, MOLT-4, HEp-2, NCI-H292, HT-29, and MCF-7 cancer cell lines, and was more cytotoxic for the HL-60 cell line, with an IC_50_ value of 11.38 μM. It also strongly inhibited the CK1δ/ε kinase.

**Scheme 24:**
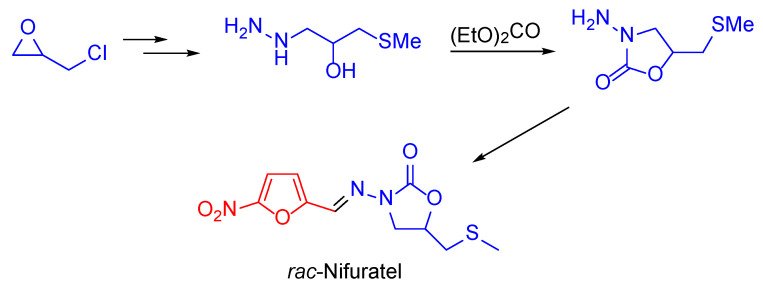
Synthesis of *rac*-nifuratel.

**Nifuratel** in its racemic form is used in gynecology as an antiprotozoal and antifungal agent. It has been reported to have a broad antibacterial spectrum of activity [71]. Several methods for its synthesis suitable for large-scale industrial production were elaborated and patented during the past decade [72,73,74]. Its general synthetic scheme is presented in Scheme 24.

The target compound was obtained at a high yield with excellent purity.

**Scheme 25:**
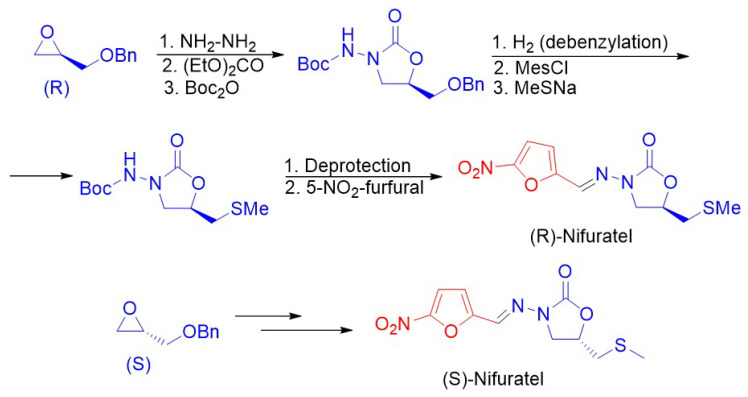
Synthesis of enantiomerically pure (*R*)- and (*S*)-nifuratel.

Optically pure (*R*)-nifuratel was found to possess a better antimicrobial activity than (*S*)-enantiomer or racemate [75]. The authors reported on the synthesis of both (*R*)- and (*S)*-nifuratel starting with the corresponding oxiranes. This method allows the resolution of the racemic compound to be avoided (Scheme 25).

### 3.2. 2-Nitroimidazoles

**Scheme 26:**
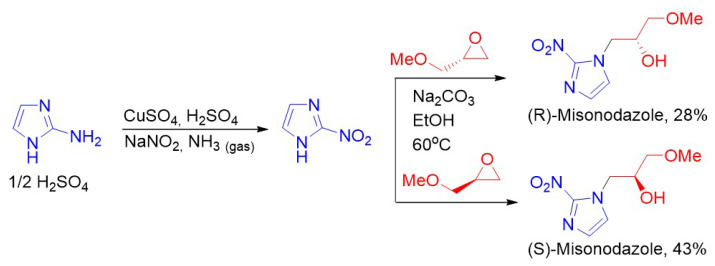
Synthesis of (*R*)- and (*S*)-misonodazole.

**Misonidazole** was considered as a radiosensitizer used in radiotherapy to cause normally resistant hypoxic tumor cells to become sensitive to the treatment [76]. It was also reported to possess potent inhibitory activity against glutathione peroxidase (GPX) from mouse liver [77]. The above-mentioned properties relate to racemic misonidazole, while two (*R*)- and (*S*)-enantiomers were synthesized independently and tested in vitro on bovine erythrocyte GPx-1 [78]. Synthesis was started with 2-aminoimidazole hemisulfate using enentiomerically pure epoxides in the second step (Scheme 26).

However, the authors did not detect any significant inhibitory activity on the bovine enzyme for either isomer.

**Scheme 27:**
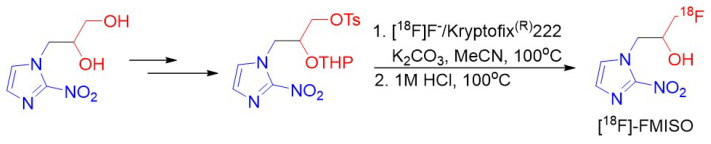
Synthesis of FMISO.

A fluorinated analog of misonidazole, namely **fluoromisanidazole** (FMISO) in its ^18^F-labeled form, is used as a radiopharmaceutical for PET imaging of hypoxia [79]. A number of synthetic approaches to ^18^F-fluoromisonidazole and its unlabeled derivative have been developed during the last decade. Several 1-alkyl-2-nitroimidazoles were used as starting materials, such as 1-(2,3-dihydroxypropyl)-2-nitroimidazole [80] (Scheme 27).

A similar fluorination procedure was applied to the intermediates bearing other protective groups instead of THP, such as acetyl [81], TBDMS, and ethoxyethyl [82].

Preparation of enantiomerically enriched [^18^F]FMISO via transition-metal-mediated enantioselective radiofluorination of epoxides and further kinetic resolution was also reported [83,84].

**Scheme 28:**
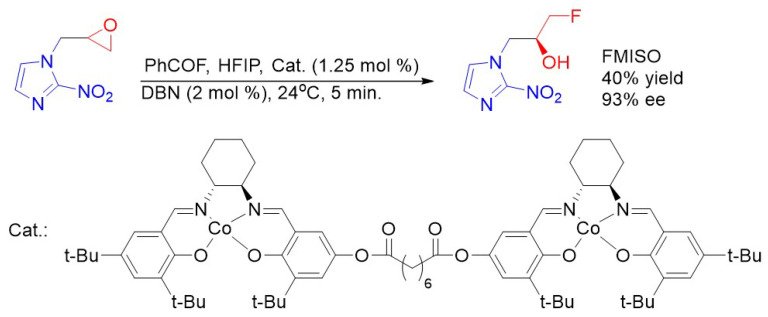
Synthesis of unlabeled FMISO.

A route excluding the deprotection step was reported by Doyle et al. [85]. An enantioselective ring opening of a nitroimidazole-substituted epoxide with benzoyl fluoride/HFIP as a fluorine source in the presence of a (salen)Co catalyst and DBN gave unlabeled FMISO at a 40% yield and 93% ee (Scheme 28).

**Scheme 29:**
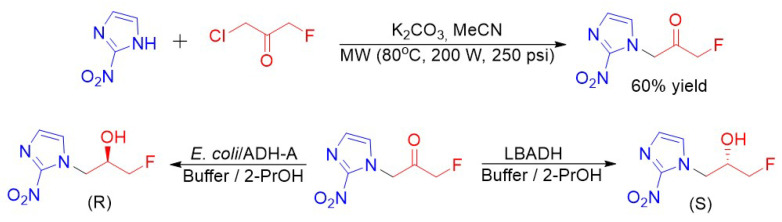
Borzecka’s synthesis of enantiopure (*R*)- and (*S*)-fluoromesonidazole.

Another efficient approach to both enantiopure (*R*)- and (*S*)-fluoromesonidazole was described by Borzecka et al. [86]. The first step uses microwave-assisted alkylation of 2-nitroimidazole with 1-chloro-3-fluoropropan-2-one to give intermediate fluoroketone. The ketone was involved in the bioreduction catalyzed by two alcohol dehydrogenases: from *Lactobacillus brevis* (LBADH) and *Escherichia coli* (E. coli/ADH-A), respectively affording enantiopure (*S*)- and (*R*)-FMISO (Scheme 29).

**Scheme 30:**
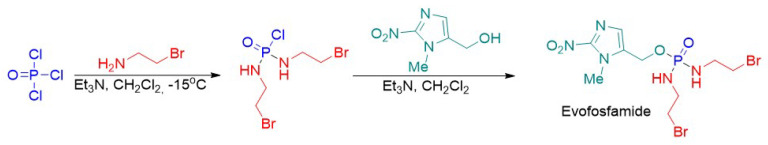
Synthesis of evofosfamide.

**Evofosfamide,** one more 2-nitroimidazole derivative, is a hypoxia-activated prodrug that is considered for cancer treatment. It was synthesized, and its cytotoxicity was evaluated in PC-3 and DU145 human prostate cancer cell lines [87]. Reaction of phosphorus oxychloride with 2-bromoethylamine resulted in intermediate dibromide, which upon reaction with 5-hydroxymethyl-1-methyl-2-nitroimidazole, gave evofosfamide at an 11.7% total yield (Scheme 30).

The authors found that evofosfamide demonstrated an increased cytotoxicity in both cell lines under hypoxic conditions relative to normoxic conditions, while a lower hypoxia selectivity in PC-3 cells relative to DU145 cells was revealed. A related method for the synthesis of evofosfamide has been recently reported [88]. The difference lies in the use of N,N′-bis(2-bromoethyl)phosphorodiamidic acid as an intermediate in place of the above-mentioned chloride.

### 3.3. 4(5)-Nitroimidazoles

**Scheme 31:**
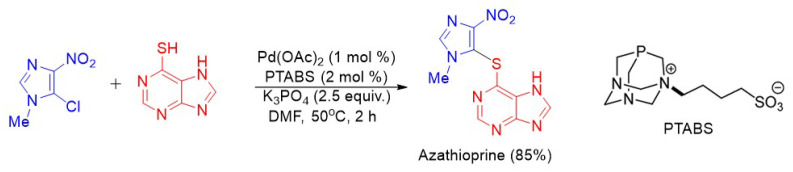
Synthesis of azathioprine.

Isomeric 4(5)-nitroimidazoles are frequently applied as efficient drugs. An example is **azathioprine** (Imuran), an immunosuppressant used in rheumatoid arthritis and many other conditions, including in kidney transplants, to prevent rejection. It was first synthesized in 1957, and since that time some efforts have been made to improve the synthetic route. The most recent publication reported on the use of a Pd/PTABS (7-phospha-1,3,5-triaza-admantane butane sulfonate) system to link thiopurine to the nitroimidazole moiety [89] (Scheme 31).

**Scheme 32:**
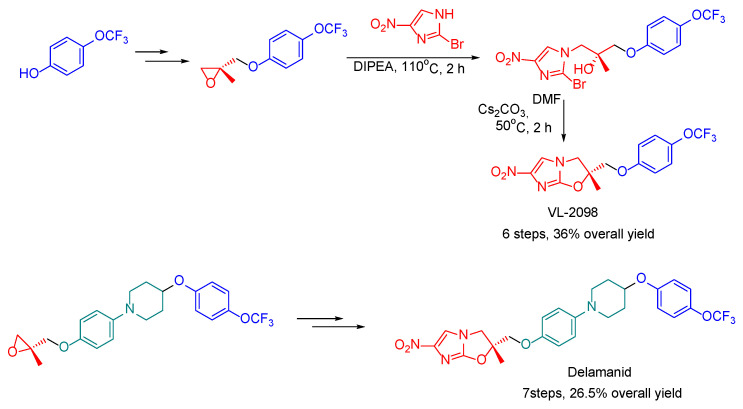
Sharma’s method for the synthesis of delamanid.

An efficient method for the synthesis of the antituberculosis drug **delamanid** and VL-2098, an antileishmanial lead candidate, was proposed by Sharma and coworkers [90]. These two related compounds incorporate a nitroimidazooxazole skeleton, which can be constructed via the nucleophilic ring opening of the chiral epoxide followed by intramolecular base-promoted cyclization (Scheme 32).

The elaborated strategy avoids the use of protecting groups and provides the opportunity for the synthesis of opposite enantiomers. Several other routes to (*R*)- and (*S*)-delamanid and their analogs using chiral intermediates have been reported recently by the same authors [91] and others [92].

**Scheme 33:**
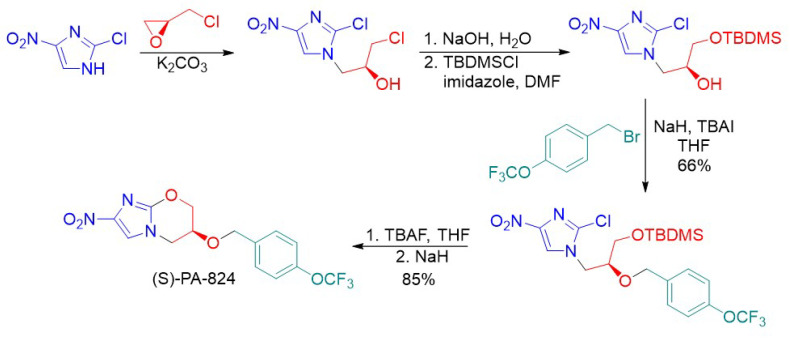
Synthetic scheme for pretomanid.

Another antibiotic medication used for the treatment of drug-resistant tuberculosis is known as **pretomanid** (PA-824). A new efficient and sustainable protocol for the synthesis of PA-824 was developed in 2020 [93]. The interaction of 2-chloro-4(5)-nitroimidazole with (S)-epichlorohydrin followed by hydrolysis and primary alcohol protection gave the key intermediate. Further O-alkylation, deprotection, and base-mediated cyclization afforded the (*S*)-enantiomer of PA-824 at a high yield (Scheme 33).

**Scheme 34:**
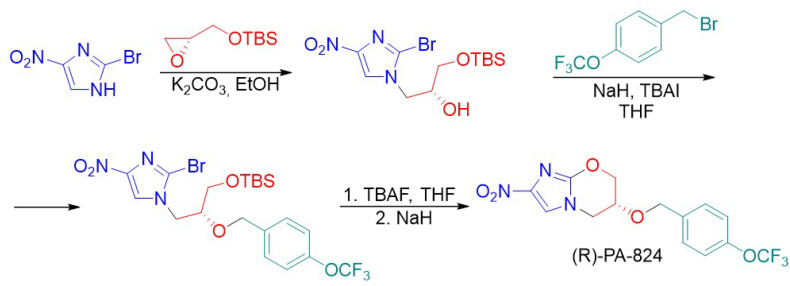
Synthesis of (*R*)-PA-824.

On the other hand, (R)-PA-824 was found to be inactive against M. tuberculosis, but showed potent activity against Leishmania donovani. The (*R*)-isomer was found to be 6-fold more active than (*S*)-PA-824 [94]. The synthesis was accomplished using a sequence similar to the one indicated above: starting with chiral O-protected epoxide and 2-bromo-4(5)-nitroimidazole, the intermediate secondary alcohol was involved in alkylation, deprotection, and base-promoted intramolecular cyclization to give the target compound at a 7% total yield (Scheme 34).

**Scheme 35:**
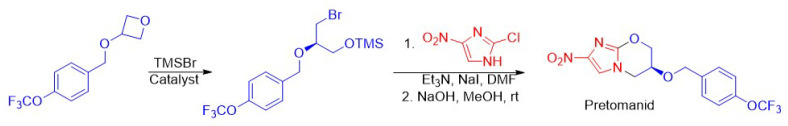
Gram-scale enantioselective synthesis of pretomanid.

Gram-scale synthesis of pretomanid has been developed using a highly enantioselective addition of TMSBr to 3-alksoxyoxetan promoted by a chiral squaramide catalyst [95]. The resulting bromide was then reacted with 2-chloro-4-nitroimidazole to give an N-alkylation product, which was deprotected and cyclized in a one-pot procedure. The total yield of the recrystallized product was 34% over three steps (Scheme 35).

Other related examples of the synthesis of PA-824 and its analogs also have been reported [96,97].

**Scheme 36:**
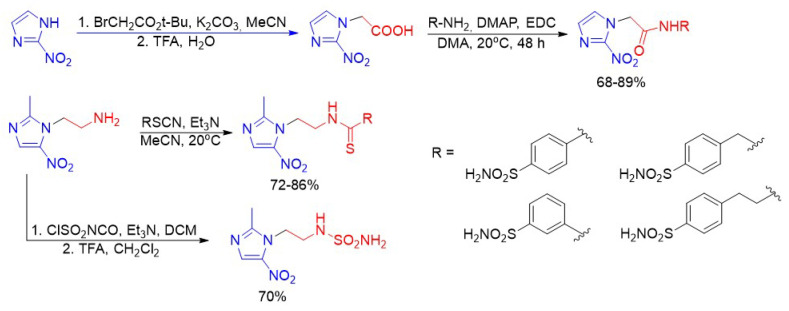
Synthesis of new class of membrane-associated carbonic anhydrase IX (CA IX) inhibitors.

A new class of membrane-associated carbonic anhydrase IX (CA IX) inhibitors containing a 2- and 5-nitroimidazole fragment along with a sulfonamide moiety was synthesized and tested in vitro by Rami and coworkers [98]. The target compounds were obtained via alkylation of 2-nitroimidazole followed by reactions with amines containing an aromatic sulfonamide group or reactions of 1-(2-aminoethyl)-2-methyl-5-nitroimidazole with rhodanobenzenes or chlorosulfonyl isocyanate (Scheme 36).

Most of the synthesized compounds were found to inhibit CA IX and XII isoforms in nanomolar concentrations.

**Scheme 37:**
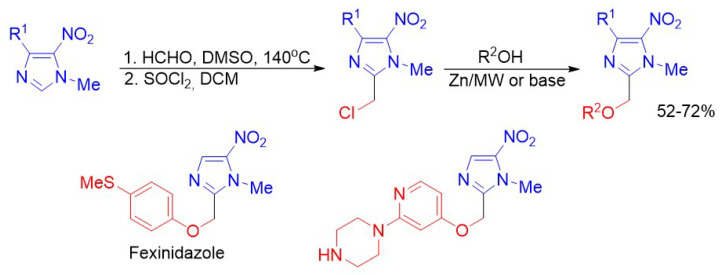
Synthesis of fexinidazole and its analogs.

**Fexinidazole** and its structural analogs were synthesized on a basis of readily available 4(5)-nitroimidazoles [99] (Scheme 37). The compounds were tested for their activity against human African trypanosomiasis caused by *Trypanosoma brucei*. In vitro testing showed potent activity of the pyridine analog of fexinidazole against *T. brucei* with a low cytotoxicity.

**Scheme 38:**
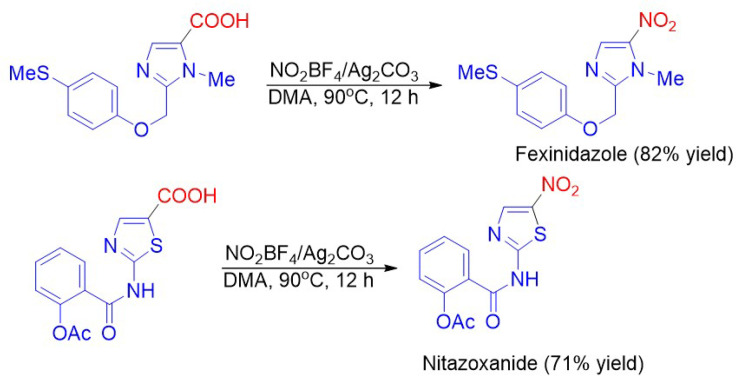
The key step in synthesis of fexinidazole and nitazoxanide.

A new and interesting synthesis of fexinidazole and **nitazoxanide** (a broad-spectrum antiparasitic and antiviral medication) was published in 2015 [100]. The key feature of this synthetic route deals with ipso-nitration of the imidazole- or thiazole-5-carboxylic acid. An efficient novel reagent representing a mixture of nitronium tetrafluoroborate and silver carbonate was used by the authors in the synthesis of a wide range of aromatic nitro compounds, including some that were pharmaceutically oriented (Scheme 38).

**Scheme 39:**
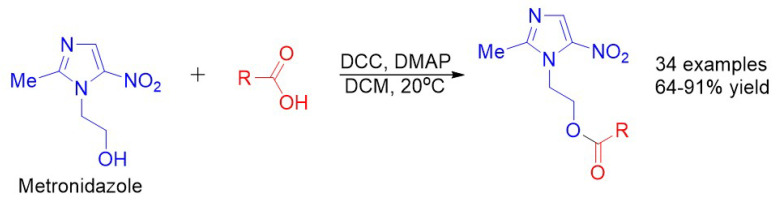
Synthesis of metronidazole derivatives.

**Metronidazole** is an antibiotic that is used for treatment of a wide range of bacterial infections, as well as trichomoniasis. Recently, Zeb et al. reported on the synthesis of metronidazole esters as a new class of antiglycation agents [101]. Reactions of metronidazole with substituted benzoic or hetarene carboxylic acids in the presence of DCC and DMAP gave corresponding esters in moderate to high yields (Scheme 39). Some of the synthesized compounds were found to be more potent as antiglycation agents than metronidazole itself.

Development of environmentally friendly methods for the synthesis of the parent metronidazole also has been reported [102,103].

**Scheme 40:**
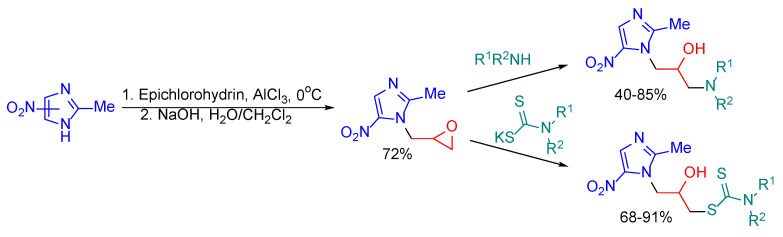
Synthesis of metronidazole analogs.

The increased resistance of *T*. *vaginalis,* which causes trichomoniasis, to metronidazole encouraged the study of new, more efficient analogs. Mandalapu and coworkers [104] synthesized a library of 4(5)-nitroimidazole derivatives and evaluated their efficacy against *T*. *vaginalis*. The synthetic scheme comprised a reaction of 2-methyl-4(5)-nitroimidazole with epichlorohydrin, epoxide formation, and its successive ring opening with a variety of substituted amines or carbamodithiolates (Scheme 40). Most of the target compounds showed higher activity as compared with metronidazole.

**Scheme 41:**
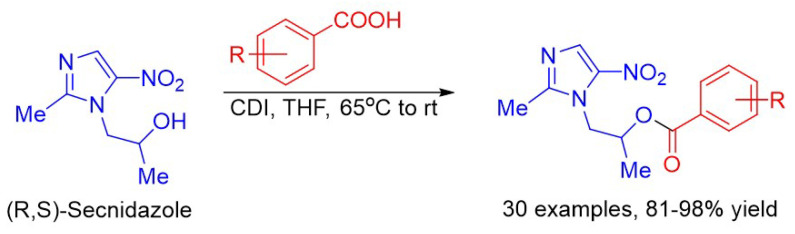
Synthesis of secnidazole esters.

**Secnidazole** is a structural analog of metronidazole used to treat vaginal infections caused by bacteria and protozoa. A series of secnidazole esters were synthesized and screened for their activities as novel enzyme inhibitors [105]. The synthesis was carried out according to a CDI-mediated coupling procedure for a wide range of substituted benzoic acids and racemic secnidazole as a partner. This protocol provided high yields of the target compounds and had an easy work-up (Scheme 41). Many of obtained compounds showed potent inhibitory activity against hCA, AChE, BChE, and α-glucosidase.

**Scheme 42:**
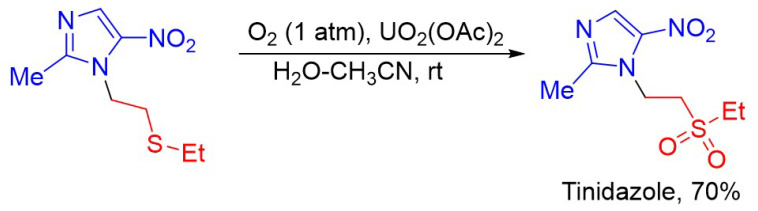
Li’s improved method for the synthesis of tinidazole.

**Tinidazole** is widely known as a treatment for a variety of anaerobic amoebic and bacterial infections. It is also a member of the nitroimidazole antibiotic class. Over the past decade, efforts have been made to improve the preparation of tinidazole salts in order to enhance their solubility and antibacterial activity [106,107]. In addition, Li and coworkers reported on tinidazole synthesis using selective late-stage oxygenation of the corresponding sulfide with ground-state oxygen under ambient conditions [108] (Scheme 42).

The above-mentioned procedure was also applied in the synthesis of other sulfoxide- and sulfone-containing pharmaceuticals, such as omeprazole, fulvestrant, sulindac, and others.

### 3.4. Miscelanous Nitroheterocycles

**Scheme 43:**
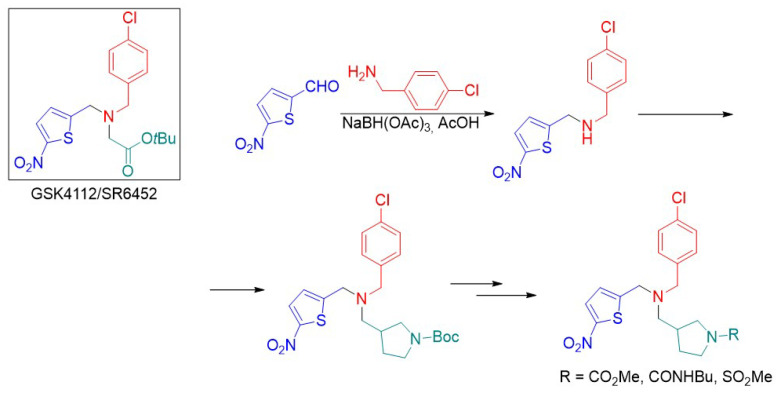
Synthesis of 5-nitrothiophene derivatives.

The nitrofurans and nitroimidazoles discussed in this chapter form the basis of heteroarene nitrodrugs. However, representatives of some other heterocyclic classes should be considered. For example, Shin et al. reported on the synthesis and structure–activity relationship study of 5-nitrothiophene derivatives [109]. A recently identified nonporphyrin synthetic ligand for Rev-erbα, GSK4112/SR6452, was taken as the lead, and a number of its analogs were synthesized via reductive amination of 5-nitrothiophene-2-carbaldehyde with 4-chlorobenzylamine in the presence of sodium triacetoxyborohydride, followed by alkylation and protective group exchange. As a result, the authors were able to greatly improve the efficacy and potency of compounds in this series when compared to GSK4112/SR6452 (Scheme 43).

**Scheme 44:**
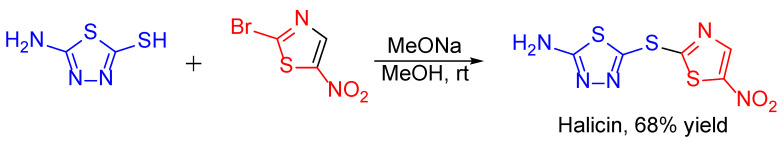
Synthesis of halicin.

**Halicin**, a 5-nitrothiazole derivative, was originally tested as an enzyme c-Jun N-terminal kinase (JNK) inhibitor [110], and was considered for treatment of diabetes; however, later it showed rather poor results. It was synthesized at a 68% yield through a nucleophilic substitution of bromine in 2-bromo5-nitrothiazole with thiol (Scheme 44).

Later, halicin was predicted as an antibacterial molecule, and showed broad-spectrum antibiotic activities in mice [111].

**Scheme 45:**
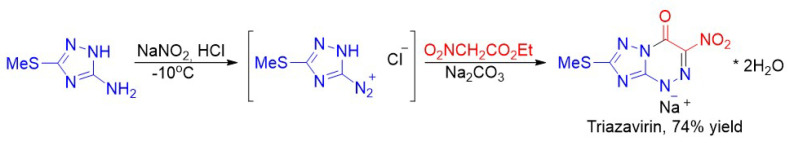
Multigram synthesis of triazavirin.

The non-nucleoside broad-spectrum antiviral drug **triazavirin** was originally developed as a potential treatment for influenza [112]. Later, it was investigated for other potential applications against virus infections. During the COVID-19 pandemic, it was tested against SARS-CoV-2 [113]. A synthesis of triazavirin was carried out at the multigram scale by Voinkov and coworkers [114] using an in situ formation of 1,2,4-triazolyl diazonium salt and its azo-coupling with ethyl nitroacetate in basic media (Scheme 45).

**Scheme 46:**
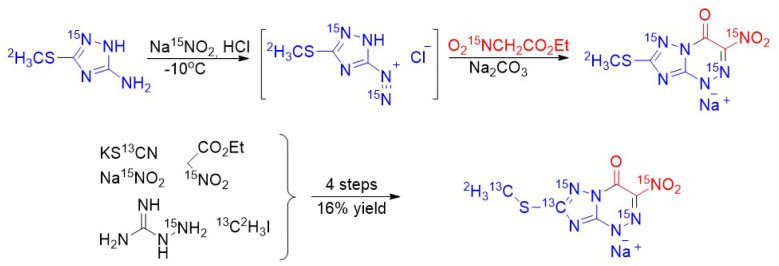
Synthesis of labeled triazavirin derivatives.

The introduction of stable isotopes such as ^2^H or ^15^N into the structures of drug candidates is an important and efficient approach to their detection by spectroscopic methods during the study of pharmacokinetics and metabolism. For this purpose, a number of labeled triazavirin molecules were synthesized recently using labeled simple starting compounds [115,116] (Scheme 46).

## 4. Conclusions

One of the goals of this review was to show the continuing interest in nitro compounds and their uses as biologically active compounds (drugs). The opinion that the nitro group negatively affects potential beneficial biological activity, and that the only possible area of application for nitro-containing molecules is compounds with special properties (explosives, energetic materials), was quite common among researchers. However, this is not the case. Many drugs that have been actively used for several decades have been created on the basis of compounds containing a nitro group. An analysis of the literature for only the last 10 years showed that the chemistry of nitro compounds is actively developing: syntheses of already-known compounds are being improved and optimized, new syntheses are being published, and new nitro-containing drugs are being created. Thus, the field of chemistry associated with the synthesis of pharmaceutically oriented molecules based on nitro compounds continues to be in demand and relevant.

## Figures and Tables

**Table 1:** Reagents and conditions.

* Conditions	Yield, %	Ref.
AcOK (2 eqviv.), DMA, 12 h, 150 °C bis{1,3-bis(2,6-diisopropylphenyl)-2,4 -diphenylimidazolium}bromobridged Pd(II) dimer	52	[20]
K_2_CO_3_, AcOH, DMA, 12 h, 130 °C 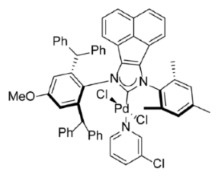	51	[21]
*t*-BuONO, MeCN, r.t. ascorbic acid (10 mol%)	41	[22]

## Data Availability

Data sharing not applicable.
